# The DREEM, part 1: measurement of the educational environment in an osteopathy teaching program

**DOI:** 10.1186/1472-6920-14-99

**Published:** 2014-05-20

**Authors:** Brett Vaughan, Annie Carter, Chris Macfarlane, Tracy Morrison

**Affiliations:** 1 College of Health & Biomedicine, Victoria University, Melbourne, Australia; 2 Institute of Sport, Exercise and Active Living, Victoria University, Melbourne, Australia

## Abstract

**Background:**

Measurement of the educational environment has become more common in health professional education programs. Information gained from these investigations can be used to implement and measure changes to the curricula, educational delivery and the physical environment. A number of questionnaires exist to measure the educational environment, and the most commonly utilised of these is the Dundee Ready Educational Environment Measure (DREEM).

**Methods:**

The DREEM was administered to students in all year levels of the osteopathy program at Victoria University (VU), Melbourne, Australia. Students also completed a demographic survey. Inferential and correlational statistics were employed to investigate the educational environment based on the scores obtained from the DREEM.

**Results:**

A response rate of 90% was achieved. The mean total DREEM score was 135.37 (+/- 19.33) with the scores ranging from 72 to 179. Some subscales and items demonstrated differences for gender, clinical phase, age and whether the student was in receipt of a government allowance.

**Conclusions:**

There are a number of areas in the program that are performing well, and some aspects that could be improved. Overall students rated the VU osteopathy program as *more positive than negative*. The information obtained in the present study has identified areas for improvement and will enable the program leaders to facilitate changes. It will also provide other educational institutions with data on which they can make comparisons with their own programs.

## Background

The educational environment has been studied across the entire spectrum from primary through to tertiary level, and even beyond to post-graduate training. Components of the educational environment include, but are not limited to: the physical infrastructure such as rooms for lectures, tutorials and clinical activities; facilitating and constraining factors for learning; the atmosphere created by fellow students; and faculty including teaching, clinical and administrative staff [1,2]. For an excellent discourse on the concepts and issues around the educational environment see Genn [3,4] who points out the environment created by a program impacts student behaviour i.e. approach to study [4,5], understanding of practice [6] and the educational outcomes achieved [3,7].

Understanding an educational program environment can assist with quality assurance by identifying where a program can be improved, and subsequently evaluating changes that are implemented [3,8,9]. Within health professional education, measurement of the environment has received some attention exploring particularly the impact on educational outcomes. Although not based on any specific educational theory [1], numerous measures of the educational environment in health professional programs have been published [10]. The most commonly utilised measure is the Dundee Ready Education Environment Measure (DREEM) [11]. Previous work by Brown et al. [12] utilised the DREEM to assess the educational environment within the allied health programs at a single Australian university (Monash University, Melbourne). These authors demonstrated a small range in total DREEM scores from 133 (Pharmacy) to 145.5 (Dietetics). As Osteopathy in Australia sits within the field of allied health, comparisons between the Monash University pre-professional programs and the current osteopathy program are appropriate. This is explored further within the discussion of the present study.

Although widely used in medical education, there is only one study in the literature adopting this measure in osteopathic education. Luciani et al. [13] employed the DREEM with the final year cohorts of three European osteopathy teaching institutions. There are currently no studies examining the educational environment of students in earlier year levels of an osteopathic curriculum, none that compare responses between year levels, nor any that investigate changes over the students’ entire time within a programme of study. The aim of the present study is to investigate the educational environment, using the DREEM, in all 5 year levels of an osteopathy program.

## Methods

This study was approved by the Victoria University Human Research Ethics Committee.

### Setting

This study was undertaken in the osteopathic discipline within the College of Health and Biomedicine at Victoria University (VU), Melbourne, Australia. The osteopathy program is five (5) years in duration with students completing a Bachelor of Science (Clinical Science) in years one to three, and a Master of Health Science in years four and five. Both degrees are required for registration as an osteopath in Australia.

### Participants

All students enrolled in the core subject Osteopathic Science were eligible to participate in the study. All students enrolled in semester two, 2013 were sent an email by the primary author (BV) informing them of the study and inviting them to participate. The email also contained a plain language statement and students were informed that all responses were anonymous and confidential.

### Measures

**Figure 1:**
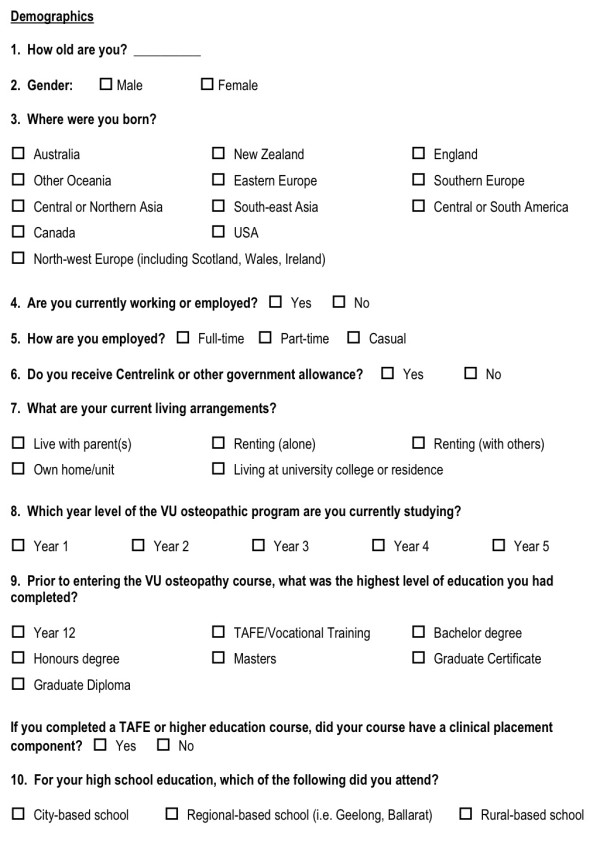
Demographic questionnaire.

Participants were invited to complete two measures: 1) a demographic questionnaire; and 2) the Dundee Ready Education Environment Measure (DREEM). The demographic questionnaire contained 11 items (Figure 1).

### DREEM

The DREEM is a 50-item questionnaire developed by Roff et al. [11] to measure the educational environment in health professional education programs. The questionnaire was developed through the use of a Delphi approach involving a range of health professional educators in different settings and different countries. As such, the DREEM is reported to be appropriate for use within health professional programs, not just medicine, and is not culture or context specific [10,14,15].

Each item is measured using a five point Likert scale: 0 is strongly disagree, 1 is disagree, 2 is neither agree or disagree, 3 is agree and 4 is strongly agree. Respondents are presented with a statement and asked to select a response. Items 4, 8, 9, 17, 25, 35, 39, 48 and 50 are negatively worded and these require recoding prior to calculating the total and subscale scores. The interpretation of the DREEM for the total score, subscales scores and item scores is presented in Table 1[2,16].

**Table 1:** Interpretation of the DREEM

**Section**	**Interpretation**
*Total DREEM score (out of 200)*	
0-50	Very poor
51-100	Plenty of problems
101-150	More positive than negative
151-200	Excellent
*DREEM subscales*	
Students’ perception of learning	0-12, very poor
	13–24, teaching is viewed negatively
	25–36, a more positive approach
	37–48, teaching highly thought of
Students’ perception of teachers	0-11, abysmal
	12–22, in need of some retraining
	23–33, moving in the right direction
	34–44, model teachers
Students’ academic self-perceptions	0-8, feeling of total failure
	9–16, many negative aspects
	17–24, feeling more on the positive side
	25–32, confident
Students’ perception of atmosphere	0-12, a terrible environment
	13–24, there are many issues that need changing
	25–36, a more positive atmosphere
	37–48, a good feeling overall
Students’ social self-perceptions	0-7, miserable
	8–14, not a nice place
	15–21, not too bad
	22–28, very good socially
*DREEM Items*	
Mean score of 3.5 or greater	Positive
Mean score between 2 and 3	Could be enhanced or improved
Mean score of 2 or less	Problematic area

The 50 items are divided into five subscales based on the initial psychometric analysis presented by Roff et al. [11]. The five subscales are Students’ Perception of Learning, Students’ Perception of Teachers, Students’ Academic Self-perceptions, Students’ Perception of Atmosphere, and Students’ Social Self-perception.

### Data collection

Students were provided with a paper-based demographic questionnaire and DREEM during the final week of the semester 2, 2013 (October 14 –18, 2013). Students were asked to complete the questionnaires at some point during the class and place it in an unmarked envelope at the front of the classroom. Students were able to submit blank questionnaires if they did not wish to be involved and completed questionnaires implied consent to participate. The envelope with the questionnaires was collected by one of the authors for analysis at the end of the class.

### Data analysis

The data from each demographic questionnaire and DREEM were manually entered into SPSS version 21 (IBM Corp, USA) by one of the authors. Mean, standard deviation and standard error of the mean (with 95% confidence intervals) were generated for each item on the DREEM along with the total and subscale scores for the DREEM, in line with the scales reported by Roff et al. [11]. The following statistics were also calculated:

1. Internal consistency of the DREEM using Cronbach’s alpha for both the total score and subscale scores as well as the alpha ‘if item deleted’;

2. Item-total correlation for each of the DREEM items;

3. Correlation between the demographic questionnaire items and the DREEM item, total and subscale scores using Pearson’s *r*; and

4. A one-way ANOVA to determine whether any difference in the DREEM total and subscale scores exists based on gender, country of birth, year level, living arrangements, education level and government allowance. Effect size calculations (Cohen’s *d*) are also presented where appropriate.

## Results

As suggested by Miles et al. [17], comprehensive statistics are presented to allow other institutions to make comparisons with the data presented here. Two hundred and forty seven responses (N = 247) from the 275 students enrolled in an Osteopathic Science subject were received representing a 90% response rate. No blank questionnaires were received. The response rates by year level were: Year 1: 75/76 (98%); Year 2: 51/56 (91%); Year 3: 34/44 (77%); Year 4: 45/54 (83%); and Year 5: 42/45 (93%). The demographic variables are presented in Table 2.

**Table 2:** Demographic data

**Age**	Mean - 23.4 years (+/- 3.99 years)
	Range – 18 to 40 years
**Gender**	Male – 115 (46.6%)
	Female – 132 (53.4%)
**Country/region of birth**	Australia – 220 (89.1%)
	South-east Asia – 7 (2.8%)
	New Zealand – 4 (1.6%)
	England – 3 (1.2%)
	Africa – 3 (1.2%)
	Other – 10 (4%)
**Currently employed**	Yes – 214 (86.6%)
	No – 33 (13.4%)
**How employed**	Full-time – 2 (0.9%)
	Part-time – 91 (42.5%)
	Casual – 121 (56.5%)
**Receive government allowance**	Yes – 118 (47.8%)
	No – 129 (52.2%)
**Current living arrangements**	Live with parents – 149 (60.3%)
	Renting with others – 76 (30.8%)
	Own home/unit – 10 (4.0%)
	Renting (alone) – 9 (3.6%)
	Living at college/residence – 3 (1.2%)
**Current year level in the osteopathy program**	Year 1 – 75 (30.4%)
	Year 2 – 51 (20.6%)
	Year 3 – 34 (13.8%)
	Year 4 – 45 (18.2%)
	Year 5 – 42 (17.0%)
**Highest level of education completed**	Year 12 – 171 (69.2%)
	Bachelor degree – 42 (17%)
	Vocational Education – 21 (8.5%)
	Higher degree – 13 (5.2%)
**Previous course with a clinical component**	Yes – 31 (13%)
	No – 215 (87%)

### Descriptive statistics

Descriptive statistics for each of the DREEM items are presented in Table 3 and descriptive statistics for each of the DREEM items by year level are presented as Additional file 1.

**Table 3:** Descriptive statistics and item-total correlations

**DREEM Item**	**Mean**	**Std. deviation**	**SEM**	**95% CI**		**Item-total correlation**
				**Lower**	**Upper**	
I am encouraged to participate in class	3.32	0.661	0.042	3.238	3.402	0.424
The course organisers are knowledgeable	3.60	0.545	0.035	3.532	3.668	0.441
There is a good support system for students who get stressed	2.40	0.896	0.057	2.288	2.512	0.416
I am too tired to enjoy this course*	1.87	1.051	0.067	1.739	2.001	0.473
Learning strategies which worked for me before continue to work for me now	2.53	0.887	0.056	2.419	2.641	0.287
The clinicians are patient with patients	2.86	0.742	0.047	2.767	2.953	0.346
The teaching is often stimulating	2.82	0.716	0.046	2.731	2.909	0.533
The teachers ridicule the students*	2.91	0.846	0.054	2.804	3.016	0.438
The teachers are authoritarian*	2.02	0.992	0.063	1.896	2.144	0.245
I am confident about passing this year	2.71	0.907	0.058	2.597	2.823	0.296
The atmosphere is relaxed during clinic teaching	2.56	0.767	0.049	2.464	2.656	0.240
This course is well timetabled	2.03	1.038	0.066	1.901	2.159	0.401
The teaching is student-centred	2.74	0.753	0.048	2.646	2.834	0.589
I am rarely bored during this course	2.30	1.020	0.065	2.173	2.427	0.480
I have good friends in this course	3.52	0.661	0.042	3.438	3.602	0.308
The teaching helps to develop my confidence	3.01	0.713	0.045	2.921	3.099	0.563
Cheating is a problem in this course*	3.06	0.909	0.058	2.947	3.173	0.187
The clinicians have good communication skills with patients	2.91	0.735	0.047	2.818	3.002	0.307
My social life is good	2.39	1.152	0.073	2.246	2.534	0.335
The teaching is well-focused	2.77	0.763	0.049	2.675	2.865	0.625
I feel I am being well prepared for my profession	2.97	0.740	0.047	2.878	3.062	0.661
The teaching helps to develop my confidence	2.87	0.730	0.046	2.779	2.961	0.662
The atmosphere is relaxed during lectures	2.89	0.663	0.042	2.807	2.973	0.377
The teaching time is put to good use	2.55	0.844	0.054	2.445	2.655	0.569
The teaching over-emphasises factual learning*	1.76	0.913	0.058	1.646	1.874	0.301
Last years work has been good preparation for this years work	2.55	0.829	0.053	2.447	2.653	0.230
I am able to memorise all I need	1.81	1.103	0.070	1.672	1.948	0.249
I seldom feel lonely	2.45	1.205	0.077	2.300	2.600	0.142
The teachers are good at providing feedback to students	2.73	0.751	0.048	2.636	2.824	0.481
There are opportunities for me to develop interpersonal skills	2.94	0.684	0.044	2.855	3.025	0.540
I have learned a lot about empathy in my profession	2.63	0.821	0.052	2.528	2.732	0.441
The teachers provide constructive criticism here	2.85	0.669	0.043	2.767	2.933	0.530
I feel comfortable in class socially	3.21	0.711	0.045	3.121	3.299	0.452
The atmosphere is relaxed during tutorials and practical session	3.06	0.631	0.040	2.981	3.139	0.438
I find the experience disappointing*	3.05	0.918	0.058	2.936	3.164	0.651
I am able to concentrate well	2.39	0.828	0.053	2.287	2.493	0.341
The teachers give clear examples	2.79	0.613	0.039	2.714	2.866	0.481
I am clear about the learning objectives of the program	2.75	0.801	0.051	2.650	2.850	0.568
The teachers get angry in class*	2.98	0.786	0.050	2.882	3.078	0.378
The teachers are well prepared for their classes	2.83	0.739	0.047	2.738	2.922	0.399
My problem solving skills are being well developed here	2.95	0.742	0.047	2.857	3.043	0.521
The enjoyment outweighs the stress of the program	2.24	1.191	0.076	2.091	2.389	0.604
The atmosphere motivates me as a learner	2.67	0.818	0.052	2.568	2.772	0.623
The teaching encourages me to be an active learner	2.82	0.793	0.050	2.721	2.919	0.622
Much of what I learn seems to be relevant to a career in osteopathy	2.92	0.942	0.060	2.803	3.037	0.521
My accommodation is pleasant	3.10	0.845	0.054	2.995	3.205	0.188
Long-term learning is emphasised over short learning	2.45	1.102	0.070	2.313	2.587	0.539
The teaching is too teacher-centred*	2.55	0.863	0.055	2.442	2.658	0.576
I feel able to ask the questions I want	3.06	0.644	0.041	2.980	3.140	0.498
The students irritate the teachers*	2.20	0.950	0.060	2.082	2.318	0.264

*Negatively worded item that requires rescoring, SEM = standard error of the mean, CI = confidence interval.

The mean DREEM total score was 135.37 (+/- 19.33) with the SEM equal to 1.235 (CI: 132.94 – 137.790). Total DREEM scores ranged from 72 to 179. The descriptive statistics for each of the five DREEM subscales are presented in Table 4.

**Table 4:** DREEM subscale statistics

**Subscale**	**Mean**	**Std. deviation**	**Subscale score interpretation**	**Alpha**
Perception of teaching	34.42	6.25	‘A more positive approach’	0.870^*^
Perception of teachers	30.69	4.25	‘Moving in the right direction’	0.703^^,#^
Academic self-perception	21.08	3.86	‘Feeling more on the positive side’	0.670
Perception of atmosphere	33.15	5.37	‘A more positive atmosphere’	0.776^%,!^
Social self-perception	18.03	3.47	‘Not too bad’	0.502^&^

Notes.

^*^Improves to 0.873 if ‘The teaching over-emphasies factual learning’ is removed.

^^^Improves to 0.712 if ‘The teachers are authoritarian’ is removed.

^#^Improves to 0.709 if ‘The students irritate the teachers’ is removed.

^%^Improves to 0.780 if ‘The atmosphere is relaxed during clinic teaching’ is removed.

^!^ Improves to 0.783 if ‘Cheating is a problem in this course’ is removed.

^&^Improves to 0.540 if ‘I seldom feel lonely’ is removed.

### Internal consistency

The Cronbach’s alpha for the DREEM was 0.923. Only the removal item 28 (I seldom feel lonely) resulted in an improvement of the alpha score to 0.925. The item-total correlations are presented in the Table 3 for the DREEM items. Table 4 also reports the ‘if item deleted’ data for the DREEM subscales.

### Relationship with demographics

#### Age

Weak, but statistically significant relationships were observed between age and:

•‘The teachers are authoritarian’ (r = 0.15, p < 0.05);

•‘I have good friends in this course’ (r = -0.17, p < 0.01);

•‘Cheating is a problem in this course’ (r = -0.24, p < 0.01);

•‘My social life is good’ (r = -0.15, p < 0.05);

•‘Last year’s work has been good preparation for this year’s work’ (r = 0.15, p < 0.05);

•‘I find the experience disappointing’ (r = -0.14, p < 0.05); and

•‘The teachers give clear examples (r = 0.18, p < 0.05).

Age was not related to the total DREEM score or subscale scores (r < 0.03).

### Highest level of education and clinical education

The highest level of education achieved by the student and whether the previous course had a clinical component was not related to the total DREEM score (r < 0.09) or subscale scores (r < 0.01).

### Between group analysis

#### Gender

No statistically significant difference was demonstrated between genders for the total DREEM score or subscale scores (p > 0.13). The effect size was d = 0.04. Statistically significant differences were noted at the item level for gender and the results are presented in Additional file 2.

#### Country of birth

There was no statistically significant difference between country of birth and the total DREEM score or subscale scores (p > 0.24).

#### Year level

Statistically significant year level differences were noted for the total DREEM score (F_(4, 242)_ = 10.24, p < 0.001) and all subscale scores (p < 0.003). Additional file 3 contains the outcome of the post-hoc testing for each year level.

#### Living arrangements

No statistically significant difference between living arrangements and the total DREEM score or subscale scores (p > 0.11) was observed.

#### Employment

There was no statistically significant difference with current employment and the total DREEM score or subscale scores (p > 0.07). There was no statistically significant difference with employment status and the total DREEM score or subscale scores (p > 0.13).

#### Government allowance

A statistically significant difference in the Social Self-Perception subscale score was demonstrated (F_(1, 245)_ = 6.00, p < 0.01). Students with a lower mean score for this subscale did not receive any form of government assistance. Further item level analysis revealed the differences to lie with the items ‘I have good friends in this course’ (F_(1, 245)_ = 4.04, p = 0.046) and ‘My social life is good’ (F_(1, 245)_ = 10.78, p = 0.001). For both items, students who were not receiving a government allowance reported lower scores on these items compared to those students who do receive an allowance. Differences for the total DREEM score and other subscales were not statistically significant (p > 0.12).

#### Clinical phase

Students were divided into pre-clinical and clinical phase groupings. Students in year 1 and 2 were classified as pre-clinical and those in year 4 and 5 were classified as clinical. Students in year 3 are in the clinical setting but only have primary patient care responsibilities late in the year. For this reason they were not included in the clinical phase of the analysis. There was no statistically significant difference for the mean total DREEM score between the phases (F_211_ = 0.178, p = 0.530). There were however statistically significant differences between the phases for the Perception of Teachers (F_211_ = 0.080, p = 0.014) and Academic self-perception subscales (F_211_ = 1.102, p <0.001). Students in the pre-clinical phase provided higher mean scores (33.02 +/- 9.35 vs 30.80 +/- 6.61) for Perception of Teachers, and students in the clinical phase provided higher mean scores for Academic self-perception (22.68 +/- 3.53 vs 19.85 +/- 3.89). Individual item differences between these groups are presented in Additional file 2.

## Discussion

To interrogate the DREEM data, Miles et al. [17] suggest the results are investigated at three levels: i) overall; ii) subscales; iii) items. Hammond et al. [18] also suggest that authors using the DREEM report the basic psychometrics.

**Figure 2:**
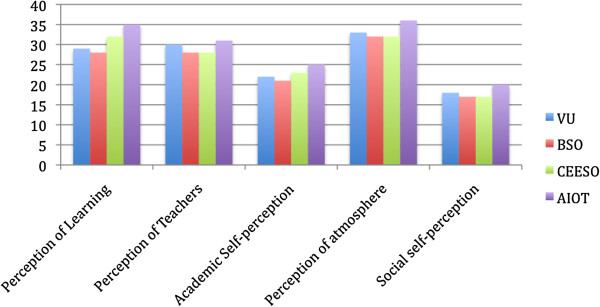
DREEM subscale comparison for final year osteopathy students.

The mean total score for the DREEM was 135 and according to the interpretation suggested by Lai et al. [19] and McAleer and Roff [16], the VU osteopathy program would be classified as *more positive than negative*. This result suggests that there are areas within the program that the students perceive as positive, but also areas requiring attention. Comparing the mean total DREEM score to those reported by Luciani et al. [13] in their study of European osteopathy programs, reveals the mean score is comparable to the British School of Osteopathy (BSO, UK) (133) and Centre Européen d'Enseignement Supérieur de l'Ostéopathie (CEESO, France) (130) but lower than Accademia Italiana Osteopatia Tradizionale (AIOT, Italy) (147). However, as these values only represent final year students, a valid approach [14] is to make a direct comparison with the final year students in the present study (Figure 2). The mean total DREEM score for year 5 students in the present study (133) again is comparable to the BSO and CEESO, as are the subscale mean scores. Luciani et al. [13] suggested the higher mean total and subscale scores at the Italian teaching institution were due to the smaller class sizes (up to 12 students), leading to more tutorial-like learning.

**Figure 3:**
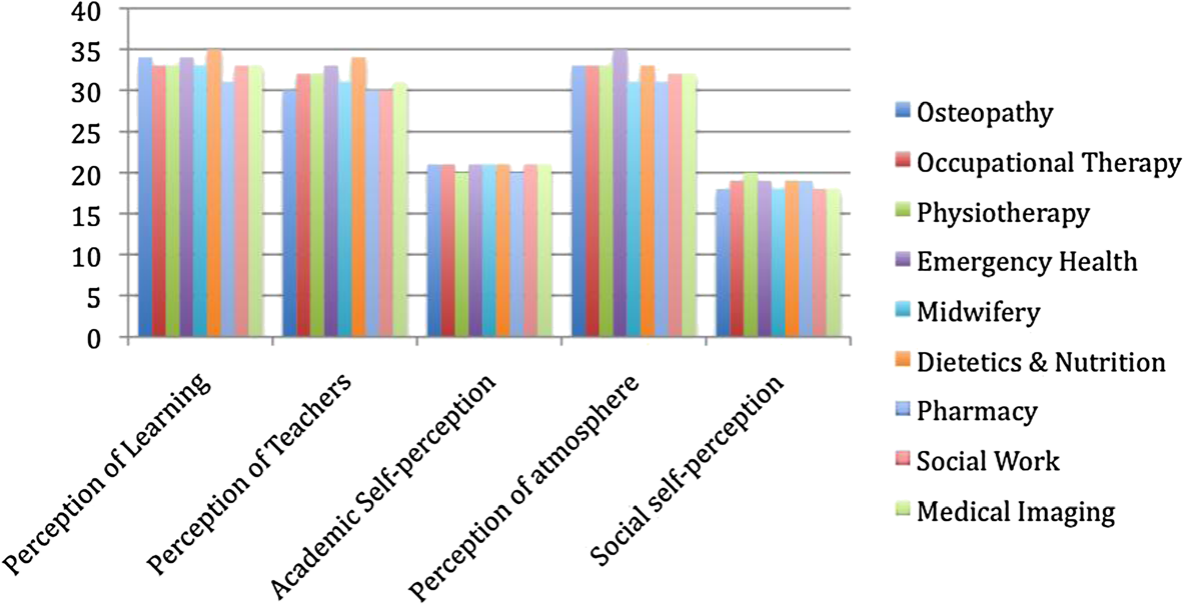
DREEM subscale comparison for programs at two Australian universities.

Figure 3 demonstrates the mean DREEM subscale scores based on the data presented by Brown et al. [12] and data from the present study. The mean total DREEM score in the present study was equal to or higher than those for Midwifery (135), Pharmacy (133), Social Work (135) and Medical Imaging (135) but lower than Physiotherapy (140), Occupational Therapy (140), Emergency Health (143) and Dietetics and Nutrition (145). The Academic self-perception subscale score is comparable across all programs. The items in this subscale reflect the student as a learner rather than the educational program per se. Subsequently, it potentially reflects the similarity in student intakes given that Monash University and Victoria University are both in metropolitan Melbourne. There are small differences in the mean scores for the other subscales however it is not possible to determine if these differences are statistically significant when comparing the osteopathy program to those allied health programs at Monash University.

### Demographics

#### Gender

With regard to the demographics and total DREEM score, there was no relationship observed for age, highest level of education achieved or whether the students’ previous program had a clinical education component. Brown et al. [12] demonstrated a statistically significant difference between females and males for the mean total DREEM score, Student perception of learning, Student perception of teaching and Student social self-perception. Females have been shown to have higher mean scores for each of the aforementioned data and this is consistent with other studies [2,11,14], particularly in Arabic countries where males and females are segregated. This gender difference was not observed in the present study (along with a negligible effect size) and concurs with previous studies [7,20-22]. As the reported effect of gender on the DREEM total and subscale score is inconsistent, this could be a topic for further investigation in different courses and contexts. At an item level, a number of items did demonstrate a difference between males and females and these are presented in Additional file 2.

#### Year level & clinical phase

**Figure 4:**
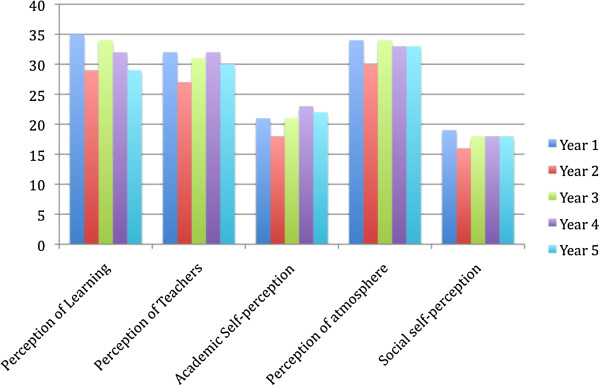
DREEM subscale scores by osteopathy program year level.

Statistically significant differences were demonstrated for the total DREEM score and all subscale scores (Figure 4). The pertinent results were the difference between the total DREEM score for year 2 students compared to all other year levels. This result was also generally reflected in the subscales, except Social self-perception. The year two student cohort has experienced a number of issues related to the teaching of the program, particularly in semester 1 and as such, the difference between year level is not unexpected. To establish whether the cohort itself accounts for the result rather than the course content or teaching, the DREEM should be re-administered at the same time in 2014.

The social self-perception subscale scores were for the most part, not statistically different between year levels. However, year 2 students had a statistically significant lower mean score for this subscale compared to those in year 1 (Additional file 3). Three items within this subscale contributed to this difference: There is a good support system for students who get stressed; I am too tired to enjoy this course; and I am rarely bored during this course. Year 2 students had lower mean scores for all of these items. Again, this result may be attributable to the reasons provided above (e.g. content, teaching) and higher teaching contact hours in year 2 compared to year 1 [23], although these reasons need to be explored further.

The difference between clinical phase and pre-clinical phase for the Academic self-perception subscale is consistent with previous research [20,21]. Students in the pre-clinical phase may perceive they are “distant from their future profession” [21] and this changes as they take on a patient-care role and can see the relevance and significance of their previous studies.

#### DREEM item responses

When examining the item level results in detail, those items with a mean of 2 or less require attention, those between 2 and 3 could be improved, and above 3 are regarded as being strong items [16,17]. Strong items and those that require attention are presented in Table 5. All other items are within the ‘could be improved’ range. Of the items suggesting areas that require attention, two would appear to be related to an emphasis on factual learning and the subsequent need to memorise this information. Numerous studies [2,21,24,25] using the DREEM have highlighted the same factual learning issue, and it has been suggested this response indicates that students are employing surface learning strategies to the detriment of deeper learning. A review of the osteopathy program is currently underway and it is anticipated that the result will include changes to the delivery of content, inclusion of peer and near-peer teaching [2,24], extension of the current portfolio [26] to year levels beyond year 5, assessments that do not emphasise factual learning and an integrated curriculum [24], all of which will assist in encouraging deeper learner engagement and improve the educational environment.

**Table 5:** Strong items and those that require improvement

**Strong items**	**Areas that require attention**
I am encouraged to participate in class	I am too tired to enjoy this course*
The course organisers are knowledgeable	The teaching over-emphasises factual learning*
I have good friends in this course	I am able to memorise all I need
The teaching helps to develop my confidence	
Cheating is a problem in this course*	
I feel comfortable in class socially	
The atmosphere is relaxed during tutorials and practical session	
I find the experience disappointing*	
My accommodation is pleasant	
I feel able to ask the questions I want	

*Negatively worded items.

Students appear to be happy with the classroom environment, both from a social and educational standpoint. This result may be attributable to the fact that the students in each year level are only ever in classes with osteopathy students in the same year; they do not have any classes at all during their program with other students at VU. The teaching staff, who are almost exclusively involved with osteopathy students, become familiar with each student creating a more personal, relaxed atmosphere in lectures and tutorials. A number of authors [27,28] have also suggested that an item about the physical environment (e.g. buildings, laboratory facilities) be added to the DREEM as these could influence the student’s overall perception.

#### Classical test theory properties of the DREEM

Hammond et al. [18] suggest that authors document the psychometric properties when reporting the use of the DREEM. The overall alpha score was α = 0.923 suggesting the measure is internally consistent for this cohort and consistent with previous research [29], however alpha scores over 0.9 can indicate item redundancy. Table 4 reports the alpha scores for each subscale. It is generally accepted that alpha scores over 0.7 indicate internal consistency. The Academic self-perception and Social self-perception subscales were below this threshold (similar to de Oliveria et al. [29] and Kossioni et al. [30]), even when an item was removed from the social subscale. It is of interest that many of the items identified for removal to improve the alpha score for the subscales were negatively worded items. It has been suggested that this type of item should not be included as the responses are variable and can adversely impact the psychometric properties of the questionnaire [31,32]. The present study did not attempt to validate the factor structure reported by the DREEM developers [11], but did use the subscales reported by these authors. A number of authors have not been able to replicate the original factor structure of the DREEM [18,20] and have subsequently questioned its internal consistency and construct validity [18]. Although comparisons have been drawn between the present study and the studies by Luciani et al. [13] and Brown et al. [12], neither reported any psychometrics in their studies. Therefore is it difficult to make any judgements about the properties of the DREEM in either an osteopathy teaching institution or Australian allied health context. Consistent with previous authors [17,18], it is suggested that those researchers using the DREEM should report: means and standard deviations for all items, subscales and the total score; effect of gender on total and subscale scores; how the items and subscales were interpreted; and internal consistency statistics for total scale and subscales. Establishing the psychometric properties of the DREEM using classical test theory and item response theory will be the subject of Part 2 in this series of papers.

Innovation and quality improvement are essential for any health professional teaching program [2] to ensure that the “…students’ learning experiences are relevant, motivating, productive, and enjoyable” [15]. The data presented in the present study is deliberately extensive in order to provide researchers in Australian allied health programs, and osteopathy programs around the world, with material/items from which they can draw comparisons with their own programs. In addition, this is the first time such extensive data using the DREEM has been presented in the health professional education literature. The VU osteopathy program intends to employ the DREEM on an ongoing basis to measure the quality of the environment subsequent to changes made to the curriculum and its delivery [33,34].

#### Strengths and limitations

The present study investigated the educational environment across a five-year osteopathy program in an Australian university. This study is the first in an Australasian osteopathy program and the first to include all year levels worldwide. A considerable strength of the present study is the high response rate ensuring the collated responses provide a reasonably accurate indication of educational environment in the VU osteopathy program. Although this study collected valuable information relating to aspects of the educational environment, we have no way of gaining a deeper understanding of the significant findings as all the data is quantitative in nature. The inclusion of some qualitative measures [35] would facilitate exploration of relevant quantitative findings.

## Conclusions

Overall, the educational environment in the osteopathy program at VU is *more positive than negative*. The environment is comparable to other Australian allied health programs and to the final year of osteopathy programs in the UK and France. Whilst the students identified a number of areas of the environment that are positive, particularly the classroom environment, there are areas in need of immediate attention. The improvements required are centred around the emphasis on factual learning and memorisation of information. The results of the present study have provided the program leaders with information that was not previously available. This information will assist them to make decisions about the action required to improve the osteopathy course at VU. It is anticipated that the DREEM will be used as part of an overarching teaching program evaluation strategy to inform reviews of the program, provide information for the program accrediting body, and measure the changes made to the program.

## Competing interests

The authors report no competing interests.

## Authors’ contributions

BV and AC devised the study. BV, AC and CM collected the data. BV undertook the data analysis. All authors contributed to the literature review and discussion. All authors approved the final version of the manuscript.

## Authors’ information

Brett Vaughan is a lecturer in the College of Health & Biomedicine, Victoria University, Melbourne, Australia and a Professional Fellow in the School of Health & Human Sciences at Southern Cross University, Lismore, New South Wales, Australia. His interests centre on competency and fitness-to-practice assessments, and clinical education in allied health.

Annie Carter is a lecturer in the College of Health & Biomedicine at Victoria University, Melbourne, Australia. Her interests include problem-based learning and student welfare issues.

Chris Macfarlane is a lecturer in the College of Health & Biomedicine at Victoria University, Melbourne, Australia. His interests include clinical education and the development of novice practitioners. He is currently completing a PhD based on the work of Dewy.

Tracy Morrison is a lecturer in the College of Health & Biomedicine at Victoria University, Melbourne, Australia. Her interests include anatomy and simulation-based education. She is currently completing her PhD on organisational change in a Australian medical school.

## Pre-publication history

The pre-publication history for this paper can be accessed here:

http://www.biomedcentral.com/1472-6920/14/99/prepub

## Supplementary Material

Additional file 1 Descriptive statistics for each DREEM item by year level. Click here for file

Additional file 2 Gender and clinical level differences. Click here for file

Additional file 3 **Between year level differences for the mean total DREEM score and each DREEM subscale score.** Social self-perception subscale analysis. Click here for file
