# Supplementary material for: The DREEM, part 1: measurement of the educational environment in an osteopathy teaching program
Source: BMC Med Educ. 2014 May 20;14:99. doi: 10.1186/1472-6920-14-99 (PMC4048620; doi:10.1186/1472-6920-14-99)
Supplement: Additional file 1 — Descriptive statistics for each DREEM item by year level. [file 1472-6920-14-99-S1.doc]

|  | | N | Mean | Std. Deviation | Std. Error | 95% Confidence Interval for Mean | | Minimum | Maximum |
| --- | --- | --- | --- | --- | --- | --- | --- | --- | --- |
|  |  |  |  |  |  | Lower Bound | Upper Bound |  |  |
| I am encouraged to participate in class | Year 1 | 75 | 3.60 | .658 | .076 | 3.45 | 3.75 | 0 | 4 |
|  | Year 2 | 51 | 3.04 | .631 | .088 | 2.86 | 3.22 | 1 | 4 |
|  | Year 3 | 34 | 3.29 | .462 | .079 | 3.13 | 3.46 | 3 | 4 |
|  | Year 4 | 45 | 3.20 | .661 | .098 | 3.00 | 3.40 | 0 | 4 |
|  | Year 5 | 42 | 3.29 | .673 | .104 | 3.08 | 3.50 | 1 | 4 |
|  | Total | 247 | 3.32 | .661 | .042 | 3.23 | 3.40 | 0 | 4 |
| The course organisers are knowledgeable | Year 1 | 75 | 3.83 | .381 | .044 | 3.74 | 3.91 | 3 | 4 |
|  | Year 2 | 51 | 3.22 | .610 | .085 | 3.04 | 3.39 | 1 | 4 |
|  | Year 3 | 34 | 3.59 | .557 | .096 | 3.39 | 3.78 | 2 | 4 |
|  | Year 4 | 45 | 3.62 | .490 | .073 | 3.47 | 3.77 | 3 | 4 |
|  | Year 5 | 42 | 3.67 | .526 | .081 | 3.50 | 3.83 | 2 | 4 |
|  | Total | 247 | 3.60 | .545 | .035 | 3.53 | 3.67 | 1 | 4 |
| There is a good support system for students who get stressed | Year 1 | 75 | 2.80 | .822 | .095 | 2.61 | 2.99 | 1 | 4 |
|  | Year 2 | 51 | 1.86 | .775 | .109 | 1.64 | 2.08 | 0 | 3 |
|  | Year 3 | 34 | 2.32 | .843 | .145 | 2.03 | 2.62 | 1 | 4 |
|  | Year 4 | 45 | 2.36 | .679 | .101 | 2.15 | 2.56 | 1 | 3 |
|  | Year 5 | 42 | 2.45 | 1.064 | .164 | 2.12 | 2.78 | 1 | 4 |
|  | Total | 247 | 2.40 | .896 | .057 | 2.29 | 2.51 | 0 | 4 |
| I am too tired to enjoy this course | Year 1 | 75 | 2.23 | 1.060 | .122 | 1.98 | 2.47 | 0 | 4 |
|  | Year 2 | 51 | 1.37 | .937 | .131 | 1.11 | 1.64 | 0 | 3 |
|  | Year 3 | 34 | 1.88 | 1.066 | .183 | 1.51 | 2.25 | 0 | 3 |
|  | Year 4 | 45 | 1.82 | .984 | .147 | 1.53 | 2.12 | 0 | 4 |
|  | Year 5 | 42 | 1.88 | 1.017 | .157 | 1.56 | 2.20 | 0 | 4 |
|  | Total | 247 | 1.87 | 1.051 | .067 | 1.74 | 2.00 | 0 | 4 |
| Learning strategies which worked for me before continue to work for me now | Year 1 | 75 | 2.55 | .827 | .095 | 2.36 | 2.74 | 1 | 4 |
|  | Year 2 | 51 | 2.43 | .922 | .129 | 2.17 | 2.69 | 0 | 4 |
|  | Year 3 | 34 | 2.62 | .739 | .127 | 2.36 | 2.88 | 1 | 4 |
|  | Year 4 | 45 | 2.64 | .857 | .128 | 2.39 | 2.90 | 1 | 4 |
|  | Year 5 | 42 | 2.45 | 1.087 | .168 | 2.11 | 2.79 | 0 | 4 |
|  | Total | 247 | 2.53 | .887 | .056 | 2.42 | 2.65 | 0 | 4 |
| The clinicians are patient with patients | Year 1 | 75 | 3.12 | .697 | .080 | 2.96 | 3.28 | 2 | 4 |
|  | Year 2 | 51 | 2.25 | .659 | .092 | 2.07 | 2.44 | 0 | 3 |
|  | Year 3 | 34 | 3.00 | .550 | .094 | 2.81 | 3.19 | 2 | 4 |
|  | Year 4 | 45 | 3.11 | .532 | .079 | 2.95 | 3.27 | 2 | 4 |
|  | Year 5 | 42 | 2.76 | .821 | .127 | 2.51 | 3.02 | 0 | 4 |
|  | Total | 247 | 2.86 | .742 | .047 | 2.77 | 2.96 | 0 | 4 |
| The teaching is often stimulating | Year 1 | 75 | 3.07 | .577 | .067 | 2.93 | 3.20 | 1 | 4 |
|  | Year 2 | 51 | 2.51 | .834 | .117 | 2.28 | 2.74 | 1 | 4 |
|  | Year 3 | 34 | 2.91 | .379 | .065 | 2.78 | 3.04 | 2 | 4 |
|  | Year 4 | 45 | 2.91 | .668 | .100 | 2.71 | 3.11 | 1 | 4 |
|  | Year 5 | 42 | 2.60 | .857 | .132 | 2.33 | 2.86 | 1 | 4 |
|  | Total | 247 | 2.82 | .716 | .046 | 2.73 | 2.91 | 1 | 4 |
| The teachers ridicule the students | Year 1 | 75 | 3.07 | .920 | .106 | 2.85 | 3.28 | 1 | 4 |
|  | Year 2 | 51 | 2.86 | .825 | .116 | 2.63 | 3.09 | 1 | 4 |
|  | Year 3 | 34 | 2.94 | .649 | .111 | 2.71 | 3.17 | 2 | 4 |
|  | Year 4 | 45 | 2.93 | .809 | .121 | 2.69 | 3.18 | 1 | 4 |
|  | Year 5 | 42 | 2.64 | .879 | .136 | 2.37 | 2.92 | 1 | 4 |
|  | Total | 247 | 2.91 | .846 | .054 | 2.80 | 3.02 | 1 | 4 |
| The teachers are authoritarian | Year 1 | 75 | 2.12 | 1.115 | .129 | 1.86 | 2.38 | 0 | 4 |
|  | Year 2 | 51 | 1.96 | .894 | .125 | 1.71 | 2.21 | 0 | 4 |
|  | Year 3 | 34 | 1.71 | 1.088 | .187 | 1.33 | 2.09 | 0 | 4 |
|  | Year 4 | 45 | 2.02 | .783 | .117 | 1.79 | 2.26 | 1 | 4 |
|  | Year 5 | 42 | 2.19 | .969 | .149 | 1.89 | 2.49 | 0 | 4 |
|  | Total | 247 | 2.02 | .992 | .063 | 1.90 | 2.15 | 0 | 4 |
| I am confident about passing this year | Year 1 | 75 | 2.55 | .920 | .106 | 2.34 | 2.76 | 0 | 4 |
|  | Year 2 | 51 | 2.24 | .862 | .121 | 1.99 | 2.48 | 0 | 4 |
|  | Year 3 | 34 | 2.56 | .860 | .147 | 2.26 | 2.86 | 1 | 4 |
|  | Year 4 | 45 | 2.78 | .636 | .095 | 2.59 | 2.97 | 1 | 4 |
|  | Year 5 | 42 | 3.64 | .485 | .075 | 3.49 | 3.79 | 3 | 4 |
|  | Total | 247 | 2.71 | .907 | .058 | 2.60 | 2.83 | 0 | 4 |
| The atmosphere is relaxed during clinic teaching | Year 1 | 75 | 2.80 | .637 | .074 | 2.65 | 2.95 | 1 | 4 |
|  | Year 2 | 51 | 2.06 | .465 | .065 | 1.93 | 2.19 | 0 | 3 |
|  | Year 3 | 34 | 2.85 | .610 | .105 | 2.64 | 3.07 | 1 | 4 |
|  | Year 4 | 45 | 2.40 | .837 | .125 | 2.15 | 2.65 | 1 | 4 |
|  | Year 5 | 42 | 2.69 | .975 | .150 | 2.39 | 2.99 | 0 | 4 |
|  | Total | 247 | 2.56 | .767 | .049 | 2.47 | 2.66 | 0 | 4 |
| This course is well timetabled | Year 1 | 75 | 2.20 | 1.053 | .122 | 1.96 | 2.44 | 0 | 4 |
|  | Year 2 | 51 | 1.78 | 1.083 | .152 | 1.48 | 2.09 | 0 | 3 |
|  | Year 3 | 34 | 2.18 | 1.167 | .200 | 1.77 | 2.58 | 0 | 4 |
|  | Year 4 | 45 | 2.04 | .952 | .142 | 1.76 | 2.33 | 0 | 4 |
|  | Year 5 | 42 | 1.88 | .889 | .137 | 1.60 | 2.16 | 0 | 3 |
|  | Total | 247 | 2.03 | 1.038 | .066 | 1.90 | 2.16 | 0 | 4 |
| The teaching is student-centred | Year 1 | 75 | 3.03 | .592 | .068 | 2.89 | 3.16 | 2 | 4 |
|  | Year 2 | 51 | 2.47 | .758 | .106 | 2.26 | 2.68 | 0 | 3 |
|  | Year 3 | 34 | 2.88 | .409 | .070 | 2.74 | 3.03 | 2 | 4 |
|  | Year 4 | 45 | 2.78 | .599 | .089 | 2.60 | 2.96 | 1 | 4 |
|  | Year 5 | 42 | 2.40 | 1.083 | .167 | 2.07 | 2.74 | 0 | 4 |
|  | Total | 247 | 2.74 | .753 | .048 | 2.65 | 2.84 | 0 | 4 |
| I am rarely bored during this course | Year 1 | 75 | 2.60 | 1.013 | .117 | 2.37 | 2.83 | 0 | 4 |
|  | Year 2 | 51 | 2.00 | .872 | .122 | 1.75 | 2.25 | 1 | 4 |
|  | Year 3 | 34 | 2.59 | .892 | .153 | 2.28 | 2.90 | 0 | 4 |
|  | Year 4 | 45 | 2.40 | .986 | .147 | 2.10 | 2.70 | 1 | 4 |
|  | Year 5 | 42 | 1.79 | 1.071 | .165 | 1.45 | 2.12 | 0 | 4 |
|  | Total | 247 | 2.30 | 1.020 | .065 | 2.17 | 2.43 | 0 | 4 |
| I have good friends in this course | Year 1 | 75 | 3.41 | .680 | .078 | 3.26 | 3.57 | 1 | 4 |
|  | Year 2 | 51 | 3.51 | .505 | .071 | 3.37 | 3.65 | 3 | 4 |
|  | Year 3 | 34 | 3.44 | .927 | .159 | 3.12 | 3.76 | 0 | 4 |
|  | Year 4 | 45 | 3.49 | .695 | .104 | 3.28 | 3.70 | 1 | 4 |
|  | Year 5 | 42 | 3.83 | .377 | .058 | 3.72 | 3.95 | 3 | 4 |
|  | Total | 247 | 3.52 | .661 | .042 | 3.44 | 3.61 | 0 | 4 |
| The teaching helps to develop my confidence | Year 1 | 75 | 3.25 | .548 | .063 | 3.13 | 3.38 | 2 | 4 |
|  | Year 2 | 51 | 2.69 | .787 | .110 | 2.46 | 2.91 | 0 | 4 |
|  | Year 3 | 34 | 3.15 | .657 | .113 | 2.92 | 3.38 | 2 | 4 |
|  | Year 4 | 45 | 3.00 | .674 | .101 | 2.80 | 3.20 | 1 | 4 |
|  | Year 5 | 42 | 2.88 | .803 | .124 | 2.63 | 3.13 | 1 | 4 |
|  | Total | 247 | 3.01 | .713 | .045 | 2.92 | 3.10 | 0 | 4 |
| Cheating is a problem in this course | Year 1 | 75 | 3.27 | .827 | .096 | 3.08 | 3.46 | 0 | 4 |
|  | Year 2 | 51 | 3.12 | .816 | .114 | 2.89 | 3.35 | 0 | 4 |
|  | Year 3 | 34 | 3.26 | .828 | .142 | 2.98 | 3.55 | 1 | 4 |
|  | Year 4 | 45 | 2.93 | .889 | .133 | 2.67 | 3.20 | 1 | 4 |
|  | Year 5 | 42 | 2.57 | 1.063 | .164 | 2.24 | 2.90 | 0 | 4 |
|  | Total | 247 | 3.06 | .909 | .058 | 2.94 | 3.17 | 0 | 4 |
| The clinicians have good communication skills with patients | Year 1 | 75 | 3.00 | .678 | .078 | 2.84 | 3.16 | 2 | 4 |
|  | Year 2 | 51 | 2.16 | .579 | .081 | 1.99 | 2.32 | 0 | 3 |
|  | Year 3 | 34 | 3.18 | .459 | .079 | 3.02 | 3.34 | 2 | 4 |
|  | Year 4 | 45 | 3.36 | .484 | .072 | 3.21 | 3.50 | 3 | 4 |
|  | Year 5 | 42 | 2.95 | .764 | .118 | 2.71 | 3.19 | 1 | 4 |
|  | Total | 247 | 2.91 | .735 | .047 | 2.81 | 3.00 | 0 | 4 |
| My social life is good | Year 1 | 75 | 2.52 | 1.131 | .131 | 2.26 | 2.78 | 0 | 4 |
|  | Year 2 | 51 | 2.10 | 1.100 | .154 | 1.79 | 2.41 | 0 | 4 |
|  | Year 3 | 34 | 2.53 | 1.080 | .185 | 2.15 | 2.91 | 0 | 4 |
|  | Year 4 | 45 | 2.18 | 1.134 | .169 | 1.84 | 2.52 | 0 | 4 |
|  | Year 5 | 42 | 2.62 | 1.268 | .196 | 2.22 | 3.01 | 0 | 4 |
|  | Total | 247 | 2.39 | 1.152 | .073 | 2.24 | 2.53 | 0 | 4 |
| The teaching is well-focused | Year 1 | 75 | 3.01 | .668 | .077 | 2.86 | 3.17 | 1 | 4 |
|  | Year 2 | 51 | 2.49 | .880 | .123 | 2.24 | 2.74 | 0 | 4 |
|  | Year 3 | 34 | 3.06 | .343 | .059 | 2.94 | 3.18 | 2 | 4 |
|  | Year 4 | 45 | 2.91 | .358 | .053 | 2.80 | 3.02 | 2 | 4 |
|  | Year 5 | 42 | 2.31 | 1.000 | .154 | 2.00 | 2.62 | 0 | 4 |
|  | Total | 247 | 2.77 | .763 | .049 | 2.68 | 2.87 | 0 | 4 |
| I feel I am being well prepared for my profession | Year 1 | 75 | 3.19 | .672 | .078 | 3.03 | 3.34 | 2 | 4 |
|  | Year 2 | 51 | 2.69 | .860 | .120 | 2.44 | 2.93 | 0 | 4 |
|  | Year 3 | 34 | 3.09 | .668 | .115 | 2.86 | 3.32 | 1 | 4 |
|  | Year 4 | 45 | 3.02 | .499 | .074 | 2.87 | 3.17 | 2 | 4 |
|  | Year 5 | 42 | 2.79 | .842 | .130 | 2.52 | 3.05 | 0 | 4 |
|  | Total | 247 | 2.97 | .740 | .047 | 2.88 | 3.06 | 0 | 4 |
| The teaching helps to develop my confidence | Year 1 | 75 | 3.23 | .606 | .070 | 3.09 | 3.37 | 0 | 4 |
|  | Year 2 | 51 | 2.39 | .896 | .125 | 2.14 | 2.64 | 0 | 4 |
|  | Year 3 | 34 | 2.88 | .591 | .101 | 2.68 | 3.09 | 1 | 4 |
|  | Year 4 | 45 | 2.89 | .532 | .079 | 2.73 | 3.05 | 1 | 4 |
|  | Year 5 | 42 | 2.81 | .671 | .104 | 2.60 | 3.02 | 1 | 4 |
|  | Total | 247 | 2.87 | .730 | .046 | 2.78 | 2.97 | 0 | 4 |
| The atmosphere is relaxed during lectures | Year 1 | 75 | 2.84 | .772 | .089 | 2.66 | 3.02 | 1 | 4 |
|  | Year 2 | 51 | 2.78 | .702 | .098 | 2.59 | 2.98 | 1 | 4 |
|  | Year 3 | 34 | 2.91 | .452 | .077 | 2.75 | 3.07 | 2 | 4 |
|  | Year 4 | 45 | 3.00 | .674 | .101 | 2.80 | 3.20 | 1 | 4 |
|  | Year 5 | 42 | 2.98 | .517 | .080 | 2.81 | 3.14 | 1 | 4 |
|  | Total | 247 | 2.89 | .663 | .042 | 2.81 | 2.97 | 1 | 4 |
| The teaching time is put to good use | Year 1 | 75 | 2.87 | .622 | .072 | 2.72 | 3.01 | 1 | 4 |
|  | Year 2 | 51 | 2.47 | .833 | .117 | 2.24 | 2.70 | 0 | 4 |
|  | Year 3 | 34 | 2.94 | .422 | .072 | 2.79 | 3.09 | 2 | 4 |
|  | Year 4 | 45 | 2.36 | .908 | .135 | 2.08 | 2.63 | 0 | 4 |
|  | Year 5 | 42 | 1.98 | 1.000 | .154 | 1.66 | 2.29 | 0 | 4 |
|  | Total | 247 | 2.55 | .844 | .054 | 2.44 | 2.66 | 0 | 4 |
| The teaching over-emphasises factual learning | Year 1 | 75 | 2.09 | .888 | .103 | 1.89 | 2.30 | 0 | 4 |
|  | Year 2 | 51 | 1.49 | .834 | .117 | 1.26 | 1.72 | 0 | 3 |
|  | Year 3 | 34 | 2.03 | .717 | .123 | 1.78 | 2.28 | 1 | 3 |
|  | Year 4 | 45 | 1.73 | .915 | .136 | 1.46 | 2.01 | 0 | 3 |
|  | Year 5 | 42 | 1.31 | .924 | .143 | 1.02 | 1.60 | 0 | 3 |
|  | Total | 247 | 1.76 | .913 | .058 | 1.65 | 1.88 | 0 | 4 |
| Last years work has been good preparation for this years work | Year 1 | 75 | 2.03 | .697 | .080 | 1.87 | 2.19 | 0 | 4 |
|  | Year 2 | 51 | 2.75 | .796 | .111 | 2.52 | 2.97 | 0 | 4 |
|  | Year 3 | 34 | 2.62 | .853 | .146 | 2.32 | 2.92 | 1 | 4 |
|  | Year 4 | 45 | 3.02 | .499 | .074 | 2.87 | 3.17 | 2 | 4 |
|  | Year 5 | 42 | 2.69 | .897 | .138 | 2.41 | 2.97 | 1 | 4 |
|  | Total | 247 | 2.55 | .829 | .053 | 2.45 | 2.65 | 0 | 4 |
| I am able to memorise all I need | Year 1 | 75 | 1.79 | 1.004 | .116 | 1.56 | 2.02 | 0 | 4 |
|  | Year 2 | 51 | 1.35 | 1.036 | .145 | 1.06 | 1.64 | 0 | 4 |
|  | Year 3 | 34 | 1.74 | 1.189 | .204 | 1.32 | 2.15 | 0 | 4 |
|  | Year 4 | 45 | 2.04 | 1.167 | .174 | 1.69 | 2.40 | 0 | 4 |
|  | Year 5 | 42 | 2.24 | 1.031 | .159 | 1.92 | 2.56 | 0 | 4 |
|  | Total | 247 | 1.81 | 1.103 | .070 | 1.68 | 1.95 | 0 | 4 |
| I seldom feel lonely | Year 1 | 75 | 2.33 | 1.223 | .141 | 2.05 | 2.61 | 0 | 4 |
|  | Year 2 | 51 | 2.53 | 1.084 | .152 | 2.22 | 2.83 | 0 | 4 |
|  | Year 3 | 34 | 2.21 | 1.321 | .226 | 1.75 | 2.67 | 0 | 4 |
|  | Year 4 | 45 | 2.73 | 1.136 | .169 | 2.39 | 3.07 | 0 | 4 |
|  | Year 5 | 42 | 2.48 | 1.273 | .196 | 2.08 | 2.87 | 0 | 4 |
|  | Total | 247 | 2.45 | 1.205 | .077 | 2.30 | 2.60 | 0 | 4 |
| The teachers are good at providing feedback to students | Year 1 | 75 | 2.93 | .644 | .074 | 2.79 | 3.08 | 1 | 4 |
|  | Year 2 | 51 | 2.49 | .809 | .113 | 2.26 | 2.72 | 0 | 4 |
|  | Year 3 | 34 | 2.79 | .479 | .082 | 2.63 | 2.96 | 1 | 3 |
|  | Year 4 | 45 | 2.64 | .830 | .124 | 2.40 | 2.89 | 1 | 4 |
|  | Year 5 | 42 | 2.69 | .869 | .134 | 2.42 | 2.96 | 1 | 4 |
|  | Total | 247 | 2.73 | .751 | .048 | 2.63 | 2.82 | 0 | 4 |
| There are opportunities for me to develop interpersonal skills | Year 1 | 75 | 3.01 | .647 | .075 | 2.86 | 3.16 | 1 | 4 |
|  | Year 2 | 51 | 2.69 | .707 | .099 | 2.49 | 2.89 | 1 | 4 |
|  | Year 3 | 34 | 3.09 | .514 | .088 | 2.91 | 3.27 | 2 | 4 |
|  | Year 4 | 45 | 2.96 | .673 | .100 | 2.75 | 3.16 | 1 | 4 |
|  | Year 5 | 42 | 2.95 | .795 | .123 | 2.70 | 3.20 | 1 | 4 |
|  | Total | 247 | 2.94 | .684 | .043 | 2.85 | 3.02 | 1 | 4 |
| I have learned a lot about empathy in my profession | Year 1 | 75 | 2.51 | .795 | .092 | 2.32 | 2.69 | 0 | 4 |
|  | Year 2 | 51 | 2.06 | .810 | .113 | 1.83 | 2.29 | 0 | 4 |
|  | Year 3 | 34 | 2.68 | .768 | .132 | 2.41 | 2.94 | 0 | 4 |
|  | Year 4 | 45 | 3.02 | .499 | .074 | 2.87 | 3.17 | 2 | 4 |
|  | Year 5 | 42 | 3.07 | .745 | .115 | 2.84 | 3.30 | 1 | 4 |
|  | Total | 247 | 2.63 | .821 | .052 | 2.52 | 2.73 | 0 | 4 |
| The teachers provide constructive criticism here | Year 1 | 75 | 3.01 | .557 | .064 | 2.89 | 3.14 | 2 | 4 |
|  | Year 2 | 51 | 2.49 | .857 | .120 | 2.25 | 2.73 | 1 | 4 |
|  | Year 3 | 34 | 2.97 | .460 | .079 | 2.81 | 3.13 | 2 | 4 |
|  | Year 4 | 45 | 2.87 | .548 | .082 | 2.70 | 3.03 | 2 | 4 |
|  | Year 5 | 42 | 2.86 | .718 | .111 | 2.63 | 3.08 | 1 | 4 |
|  | Total | 247 | 2.85 | .669 | .043 | 2.76 | 2.93 | 1 | 4 |
| I feel comfortable in class socially | Year 1 | 75 | 3.23 | .628 | .072 | 3.08 | 3.37 | 1 | 4 |
|  | Year 2 | 51 | 3.02 | .735 | .103 | 2.81 | 3.23 | 1 | 4 |
|  | Year 3 | 34 | 3.24 | .699 | .120 | 2.99 | 3.48 | 1 | 4 |
|  | Year 4 | 45 | 3.09 | .821 | .122 | 2.84 | 3.34 | 0 | 4 |
|  | Year 5 | 42 | 3.50 | .634 | .098 | 3.30 | 3.70 | 1 | 4 |
|  | Total | 247 | 3.21 | .711 | .045 | 3.12 | 3.30 | 0 | 4 |
| The atmosphere is relaxed during tutorials and practical session | Year 1 | 75 | 3.20 | .615 | .071 | 3.06 | 3.34 | 1 | 4 |
|  | Year 2 | 51 | 2.84 | .731 | .102 | 2.64 | 3.05 | 1 | 4 |
|  | Year 3 | 34 | 3.12 | .537 | .092 | 2.93 | 3.31 | 2 | 4 |
|  | Year 4 | 45 | 2.98 | .543 | .081 | 2.81 | 3.14 | 1 | 4 |
|  | Year 5 | 42 | 3.12 | .633 | .098 | 2.92 | 3.32 | 1 | 4 |
|  | Total | 247 | 3.06 | .631 | .040 | 2.98 | 3.14 | 1 | 4 |
| I find the experience disappointing | Year 1 | 75 | 3.24 | .786 | .091 | 3.06 | 3.42 | 1 | 4 |
|  | Year 2 | 51 | 2.61 | 1.021 | .143 | 2.32 | 2.90 | 0 | 4 |
|  | Year 3 | 34 | 3.18 | .758 | .130 | 2.91 | 3.44 | 1 | 4 |
|  | Year 4 | 45 | 3.16 | .706 | .105 | 2.94 | 3.37 | 1 | 4 |
|  | Year 5 | 42 | 3.02 | 1.158 | .179 | 2.66 | 3.38 | 0 | 4 |
|  | Total | 247 | 3.05 | .918 | .058 | 2.93 | 3.16 | 0 | 4 |
| I am able to concentrate well | Year 1 | 75 | 2.35 | .830 | .096 | 2.16 | 2.54 | 0 | 4 |
|  | Year 2 | 51 | 2.43 | .781 | .109 | 2.21 | 2.65 | 1 | 4 |
|  | Year 3 | 34 | 2.26 | .963 | .165 | 1.93 | 2.60 | 0 | 4 |
|  | Year 4 | 45 | 2.38 | .834 | .124 | 2.13 | 2.63 | 1 | 4 |
|  | Year 5 | 42 | 2.52 | .773 | .119 | 2.28 | 2.76 | 1 | 4 |
|  | Total | 247 | 2.39 | .828 | .053 | 2.28 | 2.49 | 0 | 4 |
| The teachers give clear examples | Year 1 | 75 | 2.73 | .664 | .077 | 2.58 | 2.89 | 1 | 4 |
|  | Year 2 | 51 | 2.67 | .712 | .100 | 2.47 | 2.87 | 1 | 4 |
|  | Year 3 | 34 | 3.03 | .460 | .079 | 2.87 | 3.19 | 2 | 4 |
|  | Year 4 | 45 | 2.91 | .557 | .083 | 2.74 | 3.08 | 1 | 4 |
|  | Year 5 | 42 | 2.74 | .497 | .077 | 2.58 | 2.89 | 1 | 3 |
|  | Total | 247 | 2.79 | .613 | .039 | 2.72 | 2.87 | 1 | 4 |
| I am clear about the learning objectives of the program | Year 1 | 75 | 2.96 | .556 | .064 | 2.83 | 3.09 | 1 | 4 |
|  | Year 2 | 51 | 2.47 | .833 | .117 | 2.24 | 2.70 | 0 | 4 |
|  | Year 3 | 34 | 2.94 | .649 | .111 | 2.71 | 3.17 | 1 | 4 |
|  | Year 4 | 45 | 3.11 | .573 | .085 | 2.94 | 3.28 | 1 | 4 |
|  | Year 5 | 42 | 2.19 | 1.042 | .161 | 1.87 | 2.52 | 0 | 4 |
|  | Total | 247 | 2.75 | .801 | .051 | 2.65 | 2.85 | 0 | 4 |
| The teachers get angry in class | Year 1 | 75 | 3.07 | .794 | .092 | 2.88 | 3.25 | 0 | 4 |
|  | Year 2 | 51 | 2.65 | .796 | .111 | 2.42 | 2.87 | 1 | 4 |
|  | Year 3 | 34 | 3.18 | .673 | .115 | 2.94 | 3.41 | 1 | 4 |
|  | Year 4 | 45 | 3.02 | .753 | .112 | 2.80 | 3.25 | 1 | 4 |
|  | Year 5 | 42 | 3.00 | .796 | .123 | 2.75 | 3.25 | 1 | 4 |
|  | Total | 247 | 2.98 | .786 | .050 | 2.88 | 3.07 | 0 | 4 |
| The teachers are well prepared for their classes | Year 1 | 75 | 2.79 | .703 | .081 | 2.62 | 2.95 | 1 | 4 |
|  | Year 2 | 51 | 3.00 | .663 | .093 | 2.81 | 3.19 | 1 | 4 |
|  | Year 3 | 34 | 3.03 | .577 | .099 | 2.83 | 3.23 | 2 | 4 |
|  | Year 4 | 45 | 2.78 | .765 | .114 | 2.55 | 3.01 | 1 | 4 |
|  | Year 5 | 42 | 2.62 | .909 | .140 | 2.34 | 2.90 | 0 | 4 |
|  | Total | 247 | 2.83 | .739 | .047 | 2.74 | 2.93 | 0 | 4 |
| My problem solving skills are being well developed here | Year 1 | 75 | 2.93 | .704 | .081 | 2.77 | 3.10 | 1 | 4 |
|  | Year 2 | 51 | 2.67 | .841 | .118 | 2.43 | 2.90 | 1 | 4 |
|  | Year 3 | 34 | 3.09 | .570 | .098 | 2.89 | 3.29 | 2 | 4 |
|  | Year 4 | 45 | 3.13 | .625 | .093 | 2.95 | 3.32 | 1 | 4 |
|  | Year 5 | 42 | 3.02 | .841 | .130 | 2.76 | 3.29 | 1 | 4 |
|  | Total | 247 | 2.95 | .742 | .047 | 2.86 | 3.04 | 1 | 4 |
| The enjoyment outweighs the stress of the program | Year 1 | 75 | 2.52 | .978 | .113 | 2.30 | 2.74 | 0 | 4 |
|  | Year 2 | 51 | 1.71 | 1.316 | .184 | 1.34 | 2.08 | 0 | 4 |
|  | Year 3 | 34 | 2.15 | 1.374 | .236 | 1.67 | 2.63 | 0 | 4 |
|  | Year 4 | 45 | 2.27 | 1.053 | .157 | 1.95 | 2.58 | 0 | 4 |
|  | Year 5 | 42 | 2.43 | 1.192 | .184 | 2.06 | 2.80 | 0 | 4 |
|  | Total | 247 | 2.24 | 1.191 | .076 | 2.09 | 2.39 | 0 | 4 |
| The atmosphere motivates me as a learner | Year 1 | 75 | 2.93 | .622 | .072 | 2.79 | 3.08 | 1 | 4 |
|  | Year 2 | 51 | 2.29 | 1.006 | .141 | 2.01 | 2.58 | 0 | 4 |
|  | Year 3 | 34 | 2.71 | .676 | .116 | 2.47 | 2.94 | 1 | 4 |
|  | Year 4 | 45 | 2.58 | .753 | .112 | 2.35 | 2.80 | 1 | 4 |
|  | Year 5 | 42 | 2.74 | .885 | .137 | 2.46 | 3.01 | 1 | 4 |
|  | Total | 247 | 2.67 | .818 | .052 | 2.57 | 2.77 | 0 | 4 |
| The teaching encourages me to be an active learner | Year 1 | 75 | 2.93 | .844 | .097 | 2.74 | 3.13 | 0 | 4 |
|  | Year 2 | 51 | 2.65 | .820 | .115 | 2.42 | 2.88 | 1 | 4 |
|  | Year 3 | 34 | 2.97 | .627 | .108 | 2.75 | 3.19 | 1 | 4 |
|  | Year 4 | 45 | 2.76 | .645 | .096 | 2.56 | 2.95 | 1 | 4 |
|  | Year 5 | 42 | 2.76 | .906 | .140 | 2.48 | 3.04 | 0 | 4 |
|  | Total | 247 | 2.82 | .793 | .050 | 2.72 | 2.92 | 0 | 4 |
| Much of what I learn seems to be relevant to a career in osteopathy | Year 1 | 75 | 3.19 | .783 | .090 | 3.01 | 3.37 | 1 | 4 |
|  | Year 2 | 51 | 2.45 | 1.026 | .144 | 2.16 | 2.74 | 0 | 4 |
|  | Year 3 | 34 | 3.09 | .830 | .142 | 2.80 | 3.38 | 1 | 4 |
|  | Year 4 | 45 | 3.11 | .682 | .102 | 2.91 | 3.32 | 1 | 4 |
|  | Year 5 | 42 | 2.67 | 1.162 | .179 | 2.30 | 3.03 | 0 | 4 |
|  | Total | 247 | 2.92 | .942 | .060 | 2.80 | 3.04 | 0 | 4 |
| My accommodation is pleasant | Year 1 | 75 | 3.03 | .854 | .099 | 2.83 | 3.22 | 0 | 4 |
|  | Year 2 | 51 | 3.10 | .922 | .129 | 2.84 | 3.36 | 0 | 4 |
|  | Year 3 | 34 | 2.85 | .857 | .147 | 2.55 | 3.15 | 1 | 4 |
|  | Year 4 | 45 | 3.18 | .806 | .120 | 2.94 | 3.42 | 1 | 4 |
|  | Year 5 | 42 | 3.33 | .721 | .111 | 3.11 | 3.56 | 1 | 4 |
|  | Total | 247 | 3.10 | .845 | .054 | 2.99 | 3.20 | 0 | 4 |
| Long-term learning is emphasised over short learning | Year 1 | 75 | 2.75 | .917 | .106 | 2.54 | 2.96 | 0 | 4 |
|  | Year 2 | 51 | 2.12 | 1.160 | .162 | 1.79 | 2.44 | 0 | 4 |
|  | Year 3 | 34 | 2.76 | .955 | .164 | 2.43 | 3.10 | 1 | 4 |
|  | Year 4 | 45 | 2.29 | 1.121 | .167 | 1.95 | 2.63 | 1 | 4 |
|  | Year 5 | 42 | 2.21 | 1.260 | .194 | 1.82 | 2.61 | 0 | 4 |
|  | Total | 247 | 2.45 | 1.102 | .070 | 2.31 | 2.58 | 0 | 4 |
| The teaching is too teacher-centred | Year 1 | 75 | 2.88 | .805 | .093 | 2.69 | 3.07 | 0 | 4 |
|  | Year 2 | 51 | 2.25 | .913 | .128 | 2.00 | 2.51 | 0 | 4 |
|  | Year 3 | 34 | 2.56 | .705 | .121 | 2.31 | 2.80 | 1 | 4 |
|  | Year 4 | 45 | 2.51 | .695 | .104 | 2.30 | 2.72 | 1 | 4 |
|  | Year 5 | 42 | 2.36 | 1.008 | .156 | 2.04 | 2.67 | 0 | 4 |
|  | Total | 247 | 2.55 | .863 | .055 | 2.44 | 2.66 | 0 | 4 |
| I feel able to ask the questions I want | Year 1 | 75 | 3.12 | .614 | .071 | 2.98 | 3.26 | 1 | 4 |
|  | Year 2 | 51 | 2.80 | .749 | .105 | 2.59 | 3.01 | 1 | 4 |
|  | Year 3 | 34 | 3.06 | .649 | .111 | 2.83 | 3.29 | 1 | 4 |
|  | Year 4 | 45 | 3.22 | .636 | .095 | 3.03 | 3.41 | 1 | 4 |
|  | Year 5 | 42 | 3.10 | .484 | .075 | 2.94 | 3.25 | 2 | 4 |
|  | Total | 247 | 3.06 | .644 | .041 | 2.98 | 3.14 | 1 | 4 |
| The students irritate the teachers | Year 1 | 75 | 2.47 | .920 | .106 | 2.25 | 2.68 | 0 | 4 |
|  | Year 2 | 51 | 2.06 | .904 | .127 | 1.80 | 2.31 | 1 | 4 |
|  | Year 3 | 34 | 1.59 | .988 | .169 | 1.24 | 1.93 | 0 | 4 |
|  | Year 4 | 45 | 2.58 | .753 | .112 | 2.35 | 2.80 | 1 | 4 |
|  | Year 5 | 42 | 2.00 | .911 | .141 | 1.72 | 2.28 | 0 | 4 |
|  | Total | 247 | 2.20 | .950 | .060 | 2.08 | 2.32 | 0 | 4 |
