# Supplementary material for: The DREEM, part 1: measurement of the educational environment in an osteopathy teaching program
Source: BMC Med Educ. 2014 May 20;14:99. doi: 10.1186/1472-6920-14-99 (PMC4048620; doi:10.1186/1472-6920-14-99)
Supplement: Additional file 2 — Gender and clinical level differences. [file 1472-6920-14-99-S2.doc]

| **DREEM Item** | **Gender** | **Clinical** |
| --- | --- | --- |
| I am encouraged to participate in class | 0.656 | 0.152 |
| The course organisers are knowledgeable | 0.540 | 0.491 |
| There is a good support system for students who get stressed | 0.047^ | 0.718 |
| I am too tired to enjoy this course* | 0.022^ | 0.748 |
| Learning strategies which worked for me before continue to work for me now | 0.133 | 0.744 |
| The clinicians are patient with patients | 0.080 | 0.101 |
| The teaching is often stimulating | 0.931 | 0.265 |
| The teachers ridicule the students* | 0.572 | 0.122 |
| The teachers are authoritarian* | 0.050 | 0.842 |
| I am confident about passing this year | 0.570 | <0.001% |
| The atmosphere is relaxed during clinic teaching | 0.007^ | 0.650 |
| This course is well timetabled | 0.877 | 0.641 |
| The teaching is student-centred | 0.223 | 0.049@ |
| I am rarely bored during this course | 0.352 | 0.073 |
| I have good friends in this course | 0.081 | 0.013% |
| The teaching helps to develop my confidence | 0.669 | 0.319 |
| Cheating is a problem in this course* | 0.626 | <0.001@ |
| The clinicians have good communication skills with patients | 0.276 | <0.001% |
| My social life is good | 0.886 | 0.741 |
| The teaching is well-focused | 0.323 | 0.117 |
| I feel I am being well prepared for my profession | 0.575 | 0.368 |
| The teaching helps to develop my confidence | 0.921 | 0.733 |
| The atmosphere is relaxed during lectures | 0.395 | 0.073 |
| The teaching time is put to good use | 0.686 | <0.001@ |
| The teaching over-emphasises factual learning* | 0.941 | 0.015@ |
| Last years work has been good preparation for this years work | 0.113 | <0.001% |
| I am able to memorise all I need | 0.096 | <0.001% |
| I seldom feel lonely | 0.763 | 0.259 |
| The teachers are good at providing feedback to students | 0.223 | 0.273 |
| There are opportunities for me to develop interpersonal skills | 0.301 | 0.716 |
| I have learned a lot about empathy in my profession | 0.001^#^ | <0.001% |
| The teachers provide constructive criticism here | 0.748 | 0.648 |
| I feel comfortable in class socially | 0.894 | 0.160 |
| The atmosphere is relaxed during tutorials and practical session | 0.843 | 0.907 |
| I find the experience disappointing* | 0.619 | 0.604 |
| I am able to concentrate well | 0.612 | 0.599 |
| The teachers give clear examples | 0.877 | 0.151 |
| I am clear about the learning objectives of the program | 0.030^#^ | 0.434 |
| The teachers get angry in class* | 0.604 | 0.226 |
| The teachers are well prepared for their classes | 0.172 | 0.112 |
| My problem solving skills are being well developed here | 0.809 | 0.016% |
| The enjoyment outweighs the stress of the program | 0.218 | 0.424 |
| The atmosphere motivates me as a learner | 0.722 | 0.783 |
| The teaching encourages me to be an active learner | 0.994 | 0.631 |
| Much of what I learn seems to be relevant to a career in osteopathy | 0.001^#^ | 0.828 |
| My accommodation is pleasant | 0.352 | 0.096 |
| Long-term learning is emphasised over short learning | 0.980 | 0.146 |
| The teaching is too teacher-centred* | 0.351 | 0.105 |
| I feel able to ask the questions I want | 0.691 | 0.153 |
| The students irritate the teachers* | 0.618 | 0.812 |

* negatively worded item that requires rescoring

^ statistically significant difference (p<0.05), mean score for males higher than females

# statistically significant difference (p<0.05), mean score for females higher than males

@ statistically significant difference (p<0.05), mean score for pre-clinical higher than clinical phase

% statistically significant difference (p<0.05), mean score for clinical higher than pre-clinical phase
