# Supplementary material for: The DREEM, part 1: measurement of the educational environment in an osteopathy teaching program
Source: BMC Med Educ. 2014 May 20;14:99. doi: 10.1186/1472-6920-14-99 (PMC4048620; doi:10.1186/1472-6920-14-99)
Supplement: Additional file 3 — Between year level differences for the mean total DREEM score and each DREEM subscale score. Social self-perception subscale analysis. [file 1472-6920-14-99-S3.doc]

**Total DREEM score (p-value)**

|  | **Year 1** | **Year 2** | **Year 3** | **Year 4** | **Year 5** |
| --- | --- | --- | --- | --- | --- |
| **Year 1** |  | <0.001* | 0.885 | 0.777 | 0.108 |
| **Year 2** |  |  | <0.001* | <0.001* | 0.019* |
| **Year 3** |  |  |  | 1.00 | 0.749 |
| **Year 4** |  |  |  |  | 0.764 |
| **Year 5** |  |  |  |  |  |

* statistically significant (p<0.05)

**Perception of teaching (p-value)**

|  | **Year 1** | **Year 2** | **Year 3** | **Year 4** | **Year 5** |
| --- | --- | --- | --- | --- | --- |
| **Year 1** |  | <0.001* | 0.804 | 0.022* | <0.001* |
| **Year 2** |  |  | <0.001* | 0.027* | 1.00 |
| **Year 3** |  |  |  | 0.552 | 0.001* |
| **Year 4** |  |  |  |  | 0.046* |
| **Year 5** |  |  |  |  |  |

* statistically significant (p<0.05)

**Perception of teachers (p-value)**

|  | **Year 1** | **Year 2** | **Year 3** | **Year 4** | **Year 5** |
| --- | --- | --- | --- | --- | --- |
| **Year 1** |  | <0.001* | 0.639 | 0.995 | 0.067 |
| **Year 2** |  |  | 0.003* | <0.001* | 0.043* |
| **Year 3** |  |  |  | 0.882 | 0.871 |
| **Year 4** |  |  |  |  | 0.255 |
| **Year 5** |  |  |  |  |  |

* statistically significant (p<0.05)

**Academic self-perception (p-value)**

|  | **Year 1** | **Year 2** | **Year 3** | **Year 4** | **Year 5** |
| --- | --- | --- | --- | --- | --- |
| **Year 1** |  | 0.013* | 0.851 | 0.022* | 0.061 |
| **Year 2** |  |  | 0.004* | <0.001* | 0.043* |
| **Year 3** |  |  |  | 0.499 | 0.675 |
| **Year 4** |  |  |  |  | 0.999 |
| **Year 5** |  |  |  |  |  |

* statistically significant (p<0.05)

**Perception of atmosphere (p-value)**

|  | **Year 1** | **Year 2** | **Year 3** | **Year 4** | **Year 5** |
| --- | --- | --- | --- | --- | --- |
| **Year 1** |  | <0.000* | 0.964 | 0.401 | 0.742 |
| **Year 2** |  |  | 0.007* | 0.054 | 0.017* |
| **Year 3** |  |  |  | 0.913 | 0.993 |
| **Year 4** |  |  |  |  | 0.991 |
| **Year 5** |  |  |  |  |  |

* statistically significant (p<0.05)

**Social self-perception (p-value)**

|  | **Year 1** | **Year 2** | **Year 3** | **Year 4** | **Year 5** |
| --- | --- | --- | --- | --- | --- |
| **Year 1** |  | 0.001* | 0.521 | 0.754 | 0.923 |
| **Year 2** |  |  | 0.374 | 0.111 | 0.056 |
| **Year 3** |  |  |  | 0.993 | 0.953 |
| **Year 4** |  |  |  |  | 0.998 |
| **Year 5** |  |  |  |  |  |

* statistically significant (p<0.05)

**Analysis of self-perception subscale items between Year 1 and Year 2**

|  | **Year 1**  **Mean (SD)** | **Year 2**  **Mean (SD)** | **p-value** |
| --- | --- | --- | --- |
| There is a good support system for students who get stressed | 2.80 (0.82) | 1.86 (0.77) | <0.001* |
| I am too tired to enjoy this course | 2.23 (1.06) | 1.37 (0.93) | <0.001* |
| I am rarely bored during this course | 2.60 (1.01) | 2.00 (0.87) | 0.001* |
| I have good friends in this course |  |  | 0.925 |
| My social life is good |  |  | 0.253 |
| I seldom feel lonely |  |  | 0.897 |
| My accommodation is pleasant |  |  | 0.990 |

* statistically significant (p<0.05)
